# Comparison of seven surrogate insulin resistance indexes for prediction of incident coronary heart disease risk: a 10-year prospective cohort study

**DOI:** 10.3389/fendo.2024.1290226

**Published:** 2024-01-23

**Authors:** Li Liu, Jie Peng, Ning Wang, Zhenguo Wu, Yerui Zhang, Huiliang Cui, Dejin Zang, Fanghong Lu, Xiaoping Ma, Jianmin Yang

**Affiliations:** ^1^ The Key Laboratory of Cardiovascular Remodeling and Function Research, Chinese Ministry of Education, Chinese National Health Commission and Chinese Academy of Medical Sciences, The State and Shandong Province Joint Key Laboratory of Translational Cardiovascular Medicine, Department of Cardiology, Qilu Hospital of Shandong University, Jinan, China; ^2^ Department of Geriatric Medicine, Qilu Hospital of Shandong University, Key Laboratory of Cardiovascular Proteomics of Shandong Province, Jinan, China; ^3^ Cardio-Cerebrovascular Control and Research Center, Shandong Academy of Medical Sciences, Jinan, China; ^4^ Department of Obstetrics and Gynecology, LiaoCheng People’s Hospital, Liaocheng, China

**Keywords:** insulin resistance surrogates, adiposity Indexes, coronary heart disease, Chinese visceral adiposity index, the triglyceride glucose index, predictive medicine

## Abstract

**Background:**

There were seven novel and easily accessed insulin resistance (IR) surrogates established, including the Chinese visceral adiposity index (CVAI), the visceral adiposity index (VAI), lipid accumulation product (LAP), triglyceride glucose (TyG) index, TyG-body mass index (TyG-BMI), TyG-waist circumference (TyG-WC) and TyG-waist to height ratio (TyG-WHtR). We aimed to explore the association between the seven IR surrogates and incident coronary heart disease (CHD), and to compare their predictive powers among Chinese population.

**Methods:**

This is a 10-year prospective cohort study conducted in China including 6393 participants without cardiovascular disease (CVD) at baseline. We developed Cox regression analyses to examine the association of IR surrogates with CHD (hazard ratio [HR], 95% confidence intervals [CI]). Moreover, the receiver operating characteristic (ROC) curve was performed to compare the predictive values of these indexes for incident CHD by the areas under the ROC curve (AUC).

**Results:**

During a median follow-up period of 10.25 years, 246 individuals newly developed CHD. Significant associations of the IR surrogates (excepted for VAI) with incident CHD were found in our study after fully adjustment, and the fifth quintile HRs (95% CIs) for incident CHD were respectively 2.055(1.216-3.473), 1.446(0.948-2.205), 1.753(1.099-2.795), 2.013(1.214-3.339), 3.169(1.926-5.214), 2.275(1.391-3.719) and 2.309(1.419-3.759) for CVAI, VAI, LAP, TyG, TyG-BMI, TyG-WC and TyG-WHtR, compared with quintile 1. Furthermore, CVAI showed maximum predictive capacity for CHD among these seven IR surrogates with the largest AUC: 0.632(0.597,0.667).

**Conclusion:**

The seven IR surrogates (excepted for VAI) were independently associated with higher prevalence of CHD, among which CVAI is the most powerful predictor for CHD incidence in Chinese populations.

## Background

Cardiovascular disease (CVD) ranked as the leading cause of deaths worldwide (1), which present a critical public health challenge and contribute to a severe disease burden in both developed and developing countries. According to World Health Organization (WHO), CVD also accounted for the largest proportion of all deaths in China (2, 3). Coronary heart disease (CHD) is the most common type of CVD in China and the main cause of cardiovascular deaths (4), which has become a serious threat to human health. Considering the increasing prevalence of CHD (5), traditional cardiovascular risk factors are no longer sufficient to predict the occurrence of CHD. Thus, there is an urgent need for novel indexes to make better prediction for incident CHD in China. Growing studies have linked insulin resistance (IR) with CVD and CHD (6–8), which also involves in the development of the metabolic syndrome (MetS) and type 2 diabetes mellitus (DM). However, the gold standard for the assessment of IR, the glucose clamp technique (9), is complex, invasive and expensive in clinical practice. As a result, many researches have focused on discovering simple, noninvasive and effective surrogates for IR to early identify specific populations at high risk of developing CHD. As is popularly known to all, obesity has been established as a traditional cardiovascular risk indicator for long. Of note, a previous research found that high brain insulin sensitivity associated closely with weight loss and a favorable body adiposity distribution (10). Accumulative studies demonstrated that a vicious circle existed between IR and obesity. An increase of adiposity deposition would lead to a proinflammatory state, which would trigger IR of all adipose tissue and non-adipose tissues by the endoplasmic reticulum stress (11). To make up for IR, pancreas make more insulin, which would promote the synthesis and storage of fat. Since obesity and IR were both linked with the incident CHD, we aimed to combine the two cardiovascular factors in the prediction of CHD using data from a 10-year prospective cohort study.

The following three non-traditional obesity indicators including the Chinese visceral adiposity index (CVAI), visceral adiposity index (VAI), and lipid accumulation product (LAP) were evidenced to be closely related to the glucose clamp technique (12, 13), as well as to be effective predictive indicators of CVD in Southwest China (14). The triglyceride glucose (TyG) index, calculated by triglyceride (TG) and fasting plasma glucose (FPG), has been reported as useful IR surrogates (15) in predicting CVD and CHD (16). Other IR surrogates calculated by obesity indicators and the TyG index including TyG-body mass index (TyG-BMI), TyG-waist circumference (TyG-WC) and TyG-waist to height ratio (TyG-WHtR) also showed efficacy in diagnosing the MetS (17) and DM (18).

However, among the seven IR surrogates above mentioned, the optimal one to recognize individuals at high risk of developing CHD remains contentious. Moreover, there were few researches focusing on whether the TyG-related indexes outperform the TyG index in predicting the CHD risk. Thus, in our present study, we explored the association of the IR surrogates with incident CHD, and compared the seven IR indicators, namely, CVAI, VAI, LAP, TyG, TyG-BMI, TyG-WC, and TyG-WHtR to determine which among them is the most appropriate IR surrogates for CHD in a 10-year cohort study in Eastern China.

## Methods

### Study subjects

The subjects of the present study were selected from twelve communities of Eastern China, based on a random, multi-stage and cluster sampling scheme. The randomly recruited participants were all aged 35-70 years and had lived in Eastern China for at least five years. In order to ensure that the sample is appropriate and robust enough, and to make the difference of the value of CVAI significant between the two independent groups using t-test at baseline in future analysis, we conducted an *a priori* power analysis using G-Power 3.1.9.7 software to calculate the sample and about 6364 participants were needed.

**Figure 1:**
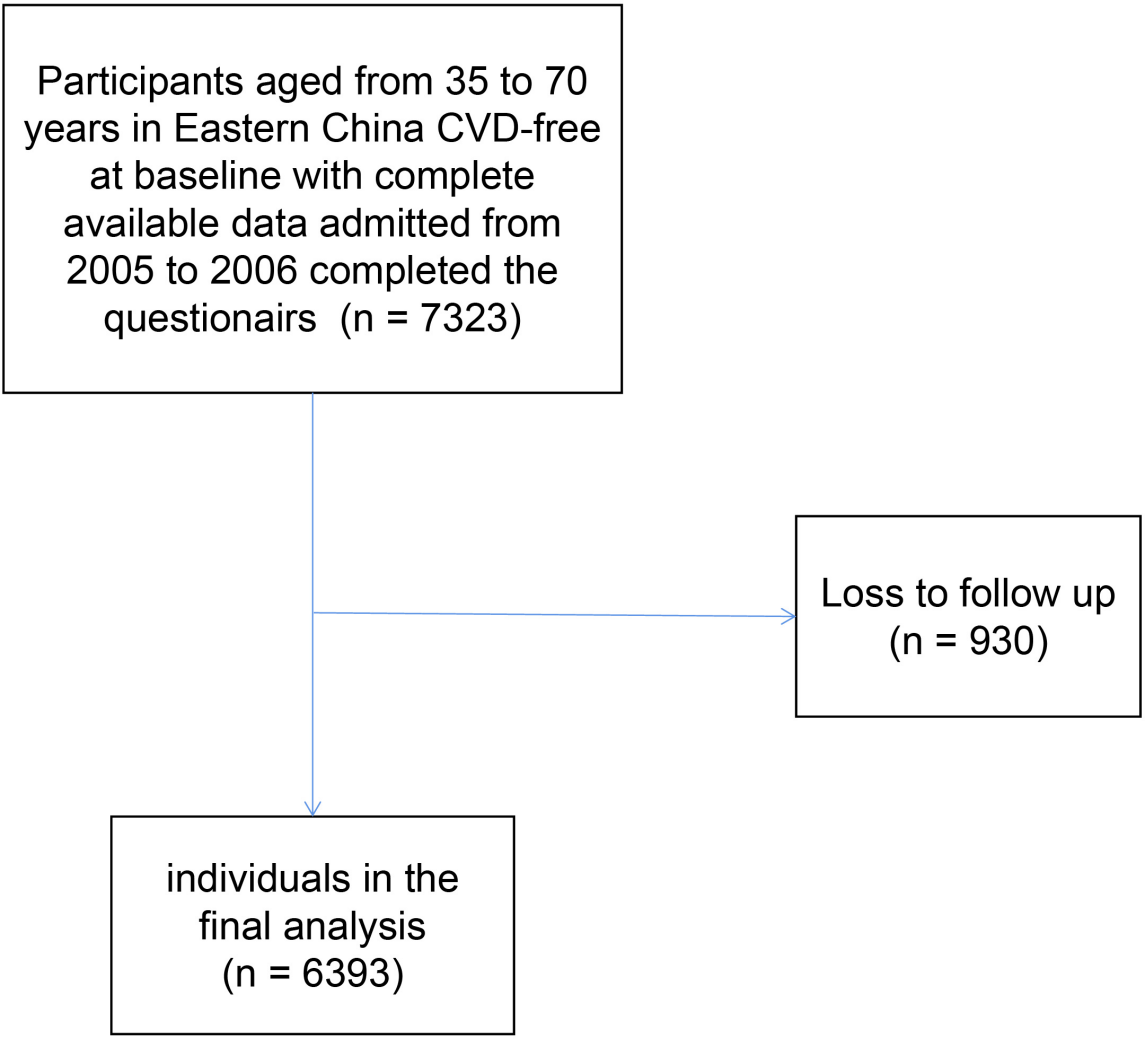
Flow diagram of patient selection. CVD, Cardiovascular disease.

In short, the present study, launched in 2005-2006, is a 10-year prospective cohort study. From 2005 to 2006, a total of 7978 participants aged 35-70 years old were included in our study. After excluding participants with CVD history at baseline or with missing data of the baseline information (n=655), there were 7323 participants available included in our investigation. With the rapid economic development and unprecedented urbanization, the migration flow in China has rapidly increased over the past two decades (19). After a 10-year follow-up, a number of 930 participants were lost, resulting in 6393 participants in the final analysis. (**Figure 1**).

### Data collection and measurement

Baseline information were collected by well-trained investigators from 2005 to 2006, through standardized interviewer-administered questionnaires. Data involving demographic factors (age, sex, education level), life styles (smoking and drinking), and individual histories of disease (DM and hypertension) were collected. Education level was categorized as primary or less, secondary, and trade, college or university. The smoking and drinking habitats were respectively divided into three categories at baseline, namely never, former and current. The International Physical Activity Questionnaire (IPAQ) was used for assessment of physical activity (20) and low physical activity was defined as < 600 metabolic equivalent task (MET)× minutes per week or < 150 min per week of moderate intensity physical activity. Anthropometric indices including weight, height and WC were measured twice, with the averages calculated. Participants were required to wear only minimal clothes and take off their shoes to get more accurate measurements by trained clinicians. Systolic blood pressure (SBP) and diastolic blood pressure (DBP) were measured three times using the sphygmomanometer with standard steps from the right arm and the averages were calculated as well. Hypertension was diagnosed according to the criteria: SBP ≥ 140mmHg or DBP ≥ 90mmHg. Ethylene Diamine Tetraacetic Acid (EDTA) anticoagulation tubes were prepared for participants fasting overnight to collect blood samples for assessment of total cholesterol (TC), TG, FPG, high-density lipoprotein cholesterol (HDL-C) and low-density lipoprotein cholesterol (LDL-C). Additionally, the outcome of our current study was CHD, defined as myocardial infarction, angina pectoris, and angiography-proven CHD. The seven IR surrogates were split into quintiles. The formulas for calculating the seven IR surrogates were published in previous studies (21–26) and calculated as follows:

BMI = weight (kg)/height^2^ (m);

WHtR = WC (cm)/height (cm);

TyG index = Ln[TG(mg/dl) x FPG (mg/dl)/2];

TyG-BMI = TyG x BMI; TyG-WC = TyG x WC; TyG-WHtR = TyG x WHtR;

Males:

CVAI=-267.93 + 0.68xage(y)+0.03xBMI(kg/m^2^)+4.00xWC(cm)+22.00xLog10TG(mmol/L)-16.32xHDL-C(mmol/L);

VAI=WC(cm)/[39.68 + 1.88xBMl(kg/m^2^)]x[1.31/HDL-C(mmol/L)]x[TG(mmol/L)/1.03];

LAP=[WC(cm)-65] x TG(mmol/L);

Females:

CVAI=-187.32 + 1.71xage(y)+4.23xBMI(kg/m^2^)+1.12xWC(cm)+39.76xLog10TG(mmol/L)-11.66xHDL-C(mmol/L);

VAI=WC(cm)/[36.58 + 1.89xBMI(kg/m^2^)]x[1.52/HDL-C(mmol/L)]x[TG(mmol/L)/0.81];

LAP=[WC(cm)-58] x TG(mmol/L).

### Statistical analysis

R software version 4.2.0 (R Foundation for Statistical Computing) was used to complete our statistical analysis and two-tailed *p* value less than 0.05 was thought significant. Participants were grouped according to the incident CHD. Continuous variables were expressed as mean ± standard deviation (SD) or median (interquartile range) and categorical variables were expressed as number (percentage) in the description of baseline characteristics. To compare the baseline data between CHD population and non-CHD population, the t-test, the Wilcoxon Mann-Whitney test and the Pearson chi-square were used for continuous and categorical variables. Each IR surrogate was categorized according to quintiles, that is, low (quintile 1), intermediate (quintiles 2-4), and high (quintile 5). The Kaplan–Meier plots and the log-rank test were used to compare the cumulative rates of incident CHD according to the quintiles. To evaluate the association between each IR surrogate and incident CHD, univariate and multivariate cox proportional hazards regression were used in our analysis. Three Cox regression models were established to clarify the predictive value of each IR surrogate: Model 1 was unadjusted; Model 2 was adjusted for age, sex, education, smoking, drinking, physical activity, TC and LDL-C; Model 3 was fully adjusted for variables in Model 2 as well as hypertension and DM. Subgroup analysis stratified by DM was conducted to further explore the association in participants with and without DM to test the stability of our results. C-statistic, net reclassification improvement (NRI) index and integrated discrimination improvement (IDI) index were calculated to estimate the incremental predictive value of each IR surrogate for incident CHD. The receiver operating characteristic (ROC) curve and the area under ROC (AUC) were used to compare the effect of CVAI, VAI, LAP, TyG, TyG-BMI, TyG-WC and TyG-WHtR on predicting the incident CHD.

## Results

### Baseline characteristics of the study population according to CHD

The baseline characteristics of the 6393 participants (50.6% were women) aged 35-70 stratified by CHD were displayed in **Table 1**. Participants with CHD were more likely to be older, former and current smokers, with higher levels of TC, TG, FPG and LDL-C, higher prevalence of hypertension and DM, and lower physical activity. Moreover, significantly increased values of obesity indexes and IR surrogates, including WC, BMI, WHtR, CVAI, VAI, LAP, TyG, TyG-BMI, TyG-WC and TyG-WHtR, were found in the CHD group (*p ≤* 0.001) (**Table 1**).

**Table 1:** Baseline characteristics of the study population according to CHD.

Variables	Total (n=6393)	Non-CHD (n=6147)	CHD (n=246)	*P-*value
Age(years)	49(17)	49(16)	56(12)	**<0.001**
Gender,n(%)				
Male	3160(49.4)	3044(49.5)	116(47.2)	0.467
Female	3233(50.6)	3103(50.5)	130(52.8)	
Smoke,n(%)				
Former	184(2.9)	169(2.7)	15(6.1)	**0.008**
Current	1310(20.5)	1258(20.5)	52(21.1)	
Never	4899(76.6)	4720(76.8)	179(72.8)	
Drink,n(%)				
Former	107(1.7)	99(1.6)	8(3.3)	0.208
Current	1305(20.4)	1254(20.4)	51(20.7)	
Never	4981(77.9)	4794(78.0)	187(76.0)	
Education,n(%)				
Primary or less	2373(37.1)	2271(36.9)	102(41.5)	0.056
Secondary	3582(56)	3461(56.3)	121(49.2)	
Trade,college or university	438(6.9)	415(6.8)	23(9.3)	
Low physical activity,n(%)	1039(16.3)	1012(16.5)	27(11.0)	**0.022**
DM,n(%)	306(4.8)	284(4.6)	22(8.9)	**0.002**
Hypertension,n(%)	3056(47.8)	2906(47.3)	150(61.0)	**<0.001**
SBP(mm Hg)	138.58 ± 21.98	138.27 ± 21.85	146.36 ± 23.75	**<0.001**
DBP(mm Hg)	84.41 ± 12.90	84.30 ± 12.89	87.09 ± 12.81	**0.001**
TC(mmol/L)	4.65 ± 0.95	4.65 ± 0.95	4.86 ± 0.93	**0.001**
TG(mmol/L)	1.19(0.98)	1.18(0.98)	1.42(1.10)	**<0.001**
FPG(mmol/L)	4.76 ± 1.53	4.75 ± 1.53	5.06 ± 1.40	**0.002**
LDL-C(mmol/L)	2.70 ± 0.75	2.69 ± 0.75	2.84 ± 0.77	**0.003**
HDL-C(mmol/L)	1.27 ± 0.32	1.27 ± 0.31	1.30 ± 0.34	0.123
WC(cm)	84.00 ± 9.22	83.90 ± 9.20	86.57 ± 9.34	**<0.001**
BMI(Kg/m^2^)	24.61 ± 3.53	24.57 ± 3.53	25.70 ± 3.57	**<0.001**
WHtR	0.51 ± 0.06	0.51 ± 0.06	0.53 ± 0.06	**<0.001**
IR surrogates				
CVAI	86.91 ± 38.45	86.23 ± 38.30	104.06 ± 38.34	**<0.001**
VAI	1.50(1.36)	1.50(1.35)	1.79(1.67)	**0.001**
LAP	26.13(31.32)	25.90(30.85)	34.55(43.33)	**<0.001**
TyG	8.44 ± 0.71	8.43 ± 0.71	8.64 ± 0.66	**<0.001**
TyG-BMI	208.61 ± 39.44	208.04 ± 39.35	222.79 ± 39.05	**<0.001**
TyG-WC	711.53 ± 114.46	710.00 ± 114.35	749.88 ± 110.65	**<0.001**
TyG-WHTR	4.35 ± 0.69	4.34 ± 0.69	4.61 ± 0.71	**<0.001**

Continuous variables were given as mean ± SD or median (interquartile range), and categorical variables were given by frequency and percentage as n(%). CHD, coronary heart disease; DM, diabetic mellitus; SBP, systolic blood pressure; DBP, diastolic blood pressure; TC, total cholesterol; TG, triglyceride; FPG, fasting plasma glucose; LDL-C, low-density lipoprotein-cholesterol; HDL-C, high-density lipoprotein-cholesterol; WC, waist circumference; BMI, body mass index; WHtR, waist-to-height ratio; IR, insulin resistance; CVAI, Chinese visceral adiposity index; VAI, visceral adiposity index; LAP, lipid accumulation product; TyG, triglyceride glucose.

*p* values in bold are < 0.05.

### Association between IR surrogates and incident CHD

**Figure 2:**
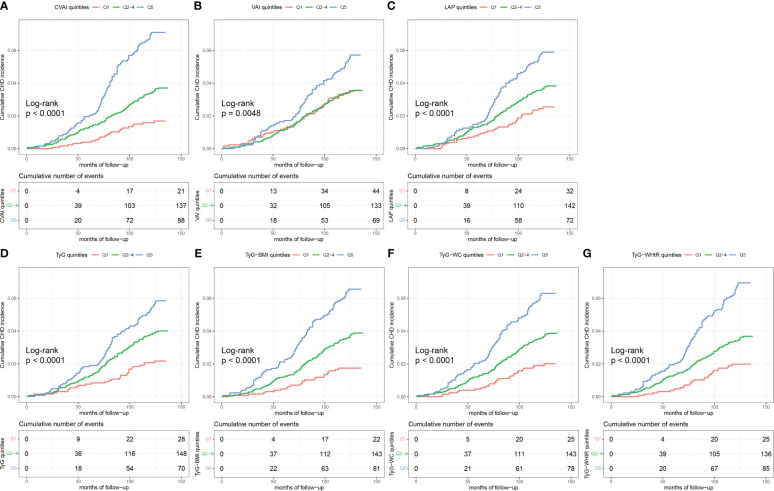
Kaplan-Meier curves of CHD by IR surrogates quintiles. The cumulative incidence of CHD during follow-up grouped according to the IR surrogates quintiles was analyzed by Kaplan-Meier curves, including CVAI **(A)**, VAI **(B)**, LAP **(C)**, TyG **(D)**, TyG-BMI **(E)**, TyG-WC **(F)**, TyG-WHtR **(G)**. The *p* value was calculated with the log-rank test. CHD, coronary heart disease; IR, insulin resistance; CVAI, Chinese visceral adiposity index; VAI, visceral adiposity index; LAP, lipid accumulation product; TyG, triglyceride glucose; BMI, body mass index; WC, waist circumference; WHtR, waist-to-height ratio.

According to **Figure 2**, the Kaplan–Meier plots showed that, marked differences in the accumulative CHD incidence were observed among the three categorized groups [low (quintile 1), intermediate (quintiles 2-4), and high (quintile 5)] of the seven IR surrogates (*p<*0.01) (**Figure 2**).

246 (3.8%) incident CHD were observed during the 10-year follow-up. **Table 2** showed the hazard ratios (HRs) and 95% confidence intervals (CIs) of incident CHD by different quintiles of IR surrogates. Compared with participants in the lowest quintile (quintile 1), those in the highest quintile (quintile 5) of CVAI, VAI, LAP, TyG, TyG-BMI, TyG-WC and TyG-WHtR had a higher risk for incident CHD after adjustment in Model 1 and Model 2, suggesting that these seven IR surrogates were all correlated with incident CHD in Model 1 and Model 2. After fully controlling the confounding factors in Model 3, with quintile 1 of IR surrogates as reference, the HRs (95%CIs) of incident CHD in quintile 5 for CVAI, VAI, LAP, TyG, TyG-BMI, TyG-WC and TyG-WHtR were respectively 2.055(1.216-3.473), 1.446(0.948-2.205), 1.753(1.099-2.795), 2.013(1.214-3.339), 3.169(1.926-5.214), 2.275(1.391-3.719) and 2.309(1.419-3.759) (all *p* < 0.05 except for VAI) (**Table 2**).

**Table 2:** HRs (95%CI) of incident CHD by quintiles of seven surrogate IR indexes.

IR surrogates		Range	Model 1	Model 2	Model 3
CVAI	Q1	≤53.41	reference	reference	reference
	Q2-4	(53.41,117.89]	2.198(1.388-3.479)***	1.487(0.926-2.389)	1.435(0.891-2.311)
	Q5	>117.89	4.385(2.724-7.058)***	2.194(1.307-3.684)**	2.055(1.216-3.473)**
	*P* for trend		**<0.001**	**0.004**	**0.010**
VAI	Q1	≤0.90	reference	reference	Reference
	Q2-4	(0.90,2.65]	1.004(0.714-1.412)	0.967(0.682-1.372)	0.938(0.660-1.332)
	Q5	>2.65	1.589(1.089-2.319)*	1.532(1.010-2.325)*	1.446(0.948-2.205)
	*P* for trend		**0.005**	**0.017**	**0.031**
LAP	Q1	≤12.67	reference	reference	Reference
	Q2-4	(12.67,53.35]	1.509(1.028-2.214)*	1.259(0.851-1.864)	1.219(0.822-1.808)
	Q5	>53.35	2.346(1.547-3.557)***	1.871(1.180-2.967)**	1.753(1.099-2.795)*
	*P* for trend		**<0.001**	**0.014**	**0.034**
TyG	Q1	≤7.87	reference	reference	reference
	Q2-4	(7.87,8.98]	1.859(1.241-2.784)**	1.487(0.981-2.252)	1.463(0.965-2.218)
	Q5	>8.98	2.691(1.736-4.171)***	2.156(1.318-3.528)**	2.013(1.214-3.339)**
	*P* for trend		**<0.001**	**0.007**	**0.024**
TyG-BMI	Q1	≤174.09	reference	reference	reference
	Q2-4	(174.09,239.34]	2.193(1.400-3.435)***	2.007(1.273-3.163)**	1.962(1.243-3.097)**
	Q5	>239.34	3.808(2.377-6.101)***	3.336(2.040-5.453)***	3.169(1.926-5.214)***
	*P* for trend		**<0.001**	**<0.001**	**<0.001**
TyG-WC	Q1	≤611.51	reference	reference	reference
	Q2-4	(611.51,803.40]	1.928(1.261-2.949)**	1.600(1.038-2.465)*	1.557(1.008-2.403)*
	Q5	>803.40	3.236(2.062-5.078)***	2.428(1.498-3.936)***	2.275(1.391-3.719)**
	*P* for trend		**<0.001**	**0.001**	**0.003**
TyG-WHtR	Q1	≤3.75	reference	reference	reference
	Q2-4	(3.75,4.89]	1.833(1.197-2.809)**	1.448(0.937-2.238)	1.408(0.909-2.181)
	Q5	>4.89	3.511(2.248-5.485)***	2.457(1.523-3.965)***	2.309(1.419-3.759)***
	*P* for trend		**<0.001**	**<0.001**	**<0.001**

Model 1:unadjusted.

Model 2: adjusted for age, sex, education, smoking, drinking, physical activity, TC, LDL-C.

Model 3: adjusted for age, sex, education, smoking, drinking, physical activity, TC, LDL-C, hypertension and DM.

HRs, hazard ratios; CI, confidence interval; CHD, coronary heart disease; IR, insulin resistance; CVAI, Chinese visceral adiposity index; VAI, visceral adiposity index; LAP, lipid accumulation product; TyG, triglyceride glucose; BMI, body mass index; WC, waist circumference; WHtR, waist-to-height ratio.

*p < 0.05.

**p <0.01.

***p < 0.001.

*P* values in bold are < 0.05.

The subgroup analysis shown in **Table 3** suggested that there were no significant association of seven IR surrogates with incident CHD in participants with DM (*p* > 0.05). While in participants without DM, per SD increase in these IR surrogates (except for VAI) was correlated with an increased risk of incident CHD, with the fully adjusted HRs (95%CI) being 1.250(1.076,1.452), 1.148(1.031,1.279), 1.341(1.128,1.594), 1.371(1.201,1.564), 1.293(1.113,1.501) and 1.281(1.105,1.486) for CVAI, LAP, TyG, TyG-BMI, TyG-WC and TyG-WHtR, respectively. (**Table 3**).

**Table 3:** HRs (95%CI) of incident CHD by per SD increase of seven baseline surrogate IR indexes and subgroup analysis according to history of DM.

Variables (per SD)	HR(95%CI) for per SD increase		
	Total	DM	Without DM
CVAI	1.233(1.068-1.423)**	0.998(0.558,1.783)	1.250(1.076,1.452)**
VAI	1.007(0.918-1.105)	1.280(0.275,5.951)	1.007(0.919,1.105)
LAP	1.097(0.986-1.220)	0.938(0.646,1.363)	1.148(1.031,1.279)*
TyG	1.283(1.095-1.503)**	0.994(0.631,1.564)	1.341(1.128,1.594)***
TyG-BMI	1.343(1.183-1.525)***	1.044(0.643,1.695)	1.371(1.201,1.564)***
TyG-WC	1.256(1.090-1.447)**	0.930(0.554,1.563)	1.293(1.113,1.501)***
TyG-WHtR	1.252(1.088-1.441)**	0.975(0.597,1.592)	1.281(1.105,1.486)**

HR are adjusted for age, sex, education, smoking, drinking, physical activity, TC, LDL-C, hypertension and DM.

HRs, hazard ratios; CI, Confidence interval; CHD, coronary heart disease; SD, standard deviation; IR, insulin resistance; DM, diabetic mellitus; CVAI, Chinese visceral adiposity index; VAI, visceral adiposity index; LAP, lipid accumulation product; TyG, triglyceride glucose; BMI, body mass index; WC, waist circumference; WHtR, waist-to-height ratio.

*p < 0.05.

**p <0.01.

***p < 0.001.

### Evaluation of the prognostic performance of each IR surrogate for incident CHD

As displayed in **Table 4**, adding the IR surrogates into the traditional baseline risk model, including age, sex, education, smoking, drinking, physical activity, TC, LDL-C, hypertension and DM, significantly improved the prediction capacity of incident CHD according to continuous NRI. The continuous NRI for CVAI, VAI, LAP, TyG, TyG-BMI, TyG-WC and TyG-WHtR were respectively 0.2431(0.1169,0.3694), 0.1389(0.0150,0.2628), 0.1668(0.0396,0.2940), 0.2451(0.1193,0.3709), 0.2546(0.1275,0.3816), 0.1927(0.0656,0.3199) and 0.2084(0.0814,0.3354) (all p < 0.05). However, IDI and C-statistics did not show statistically significance in improving the prediction of CHD for LAP and VAI (**Table 4**).

**Table 4:** The incremental predictive value of the seven IR surrogates for incident CHD.

Variables	C-statistics(95%CI)	*P*-value	Continuous NRI(95%CI)	*P*-value	IDI(95%CI)	*P*-value
Model 3 without CVAI	0.704(0.674,0.735)	Ref.		Ref.		Ref.
Model 3 with CVAI	0.708(0.677,0.739)	**0.004**	0.2431(0.1169,0.3694)	**<0.001**	0.0020(5e-04,0.0036)	**0.012**
Model 3 without VAI	0.704(0.674,0.735)	Ref.		Ref.		Ref.
Model 3 with VAI	0.704(0.674,0.735)	0.904	0.1389(0.0150,0.2628)	**0.028**	0(0, 0)	0.496
Model 3 without LAP	0.704(0.674,0.735)	Ref.		Ref.		Ref.
Model 3 with LAP	0.706(0.675,0.737)	0.130	0.1668(0.0396,0.2940)	**0.010**	5e-04(-1e-04, 0.001)	0.105
Model 3 without TyG	0.704(0.674,0.735)	Ref.		Ref.		Ref.
Model 3 with TyG	0.711(0.681,0.741)	**0.003**	0.2451(0.1193,0.3709)	**<0.001**	0.0017(1e-04,0.0032)	**0.037**
Model 3 without TyG-BMI	0.704(0.674,0.735)	Ref.		Ref.		Ref.
Model 3 with TyG-BMI	0.716(0.686,0.746)	**<0.001**	0.2546(0.1275,0.3816)	**<0.001**	0.0042(0.0018,0.0066)	**<0.001**
Model 3 without TyG-WC	0.704(0.674,0.735)	Ref.		Ref.		Ref.
Model 3 with TyG-WC	0.710(0.679,0.740)	**0.002**	0.1927(0.0656,0.3199)	**0.003**	0.0023(6e-04,0.004)	**0.010**
Model 3 without TyG-WHtR	0.704(0.674,0.735)	Ref.		Ref.		Ref.
Model 3 with TyG-WHtR	0.709(0.678,0.739)	**0.002**	0.2084(0.0814,0.3354)	**0.001**	0.0023(5e-04,0.0041)	**0.010**

IR, insulin resistance; CHD, coronary heart disease; CI, Confidence intervals; NRI, net reclassification improvement; IDI, integrated discrimination improvement; CVAI, Chinese visceral adiposity index; Ref., reference; VAI, visceral adiposity index; LAP, lipid accumulation product; TyG, triglyceride glucose; BMI, body mass index; WC, waist circumference; WHtR, waist-to-height ratio.

*P* values in bold are < 0.05.

### The predictive ability of each IR surrogate for incident CHD

**Figure 3:**
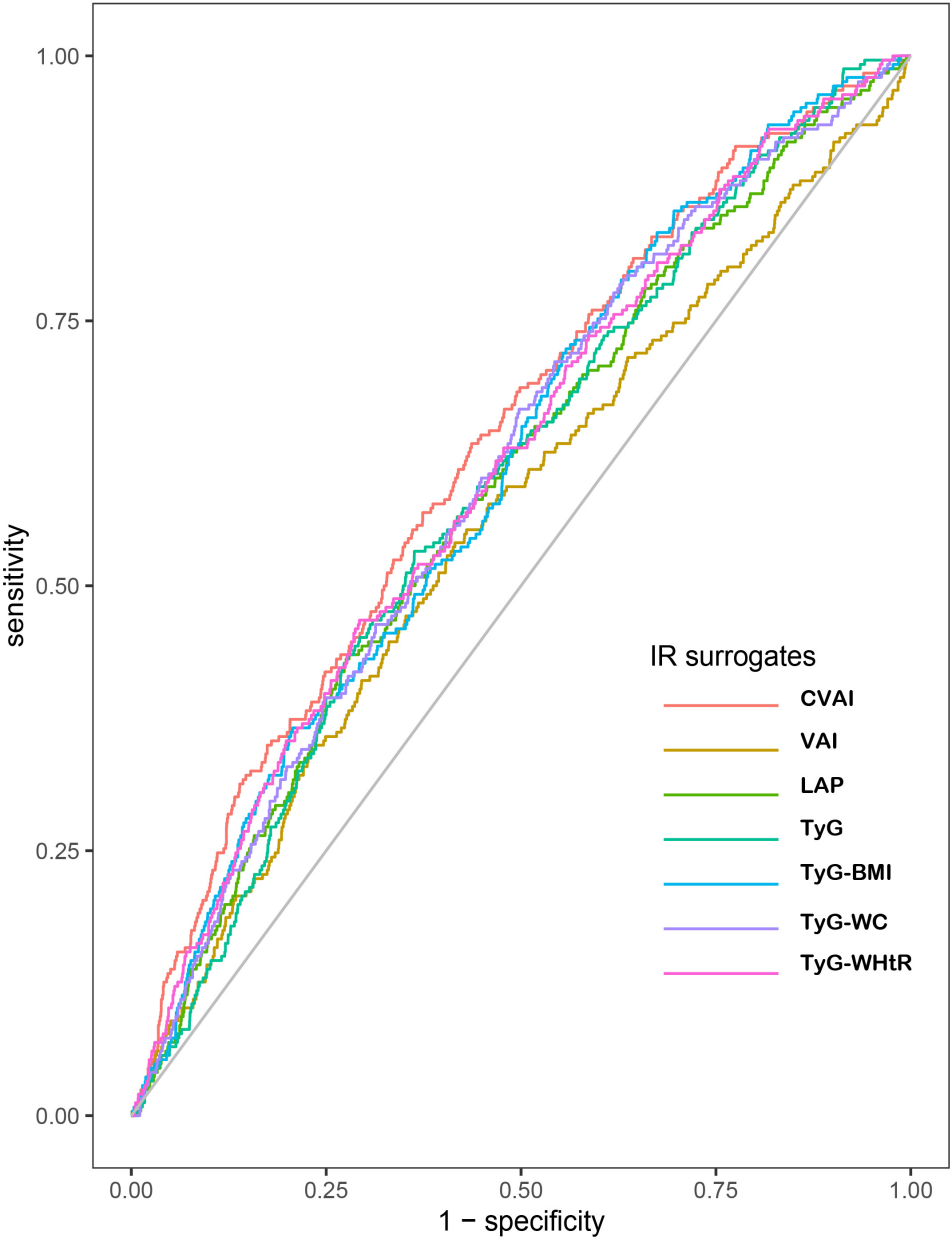
Receiver operating characteristic curves of CHD by IR surrogates quintiles. The AUCs (95%CI) of indexes are as follows: CVAI: 0.632(0.597,0.667); VAI: 0.560(0.522,0.598); LAP:0.593(0.557,0.629); TyG: 0.595(0.560,0.630); TyG-BMI: 0.609(0.574,0.644); TyG-WC: 0.606(0571,0.641); TyG-WHtR: 0.609(0.574,0.645), respectively, p<0.01. CHD, coronary heart disease; IR, insulin resistance; CVAI, Chinese visceral adiposity index; VAI, visceral adiposity index; LAP, lipid accumulation product; TyG, triglyceride glucose; BMI, body mass index; WC, waist circumference; WHtR, waist-to-height ratio.

**Table 5** and **Figure 3** showed the AUCs and ROC of IR surrogates for predicting the incident CHD. Among all IR surrogates studied in the present study, CVAI showed the optimal predictive power for incident CHD with the highest value of AUC. The AUCs and 95%CI for each IR surrogate were displayed as follows: 0.632(0.597,0.667) for CVAI, 0.560(0.522,0.598) for VAI, 0.593(0.557,0.629) for LAP, 0.595(0.560,0.630) for TyG, 0.609(0.574,0.644) for TyG-BMI, 0.606(0.571,0.641) for TyG-WC and 0.609(0.574,0.645) for TyG-WHTR respectively. According to the delong test conducted to compare the AUCs of IR surrogates, with CVAI as reference, the results suggested that CVAI had significantly different AUC from other IR surrogates (all *p* < 0.05) (**Table 5** and **Figure 3**).

**Table 5:** AUCs of seven IR surrogates for predicting CHD among study populations.

Variables	Total		Delong test	
	AUC(95%CI)	*P*-value	Z-value	*P*-value
CVAI	0.632(0.597,0.667)	**<0.001**	reference	
VAI	0.560(0.522,0.598)	**0.001**	-4.149	**<0.001**
LAP	0.593(0.557,0.629)	**<0.001**	-3.580	**<0.001**
TyG	0.595(0.560,0.630)	**<0.001**	-2.169	**0.030**
TyG-BMI	0.609(0.574,0.644)	**<0.001**	-2.001	**0.045**
TyG-WC	0.606(0.571,0.641)	**<0.001**	3.124	**0.002**
TyG-WHtR	0.609(0.574,0.645)	**<0.001**	-2.405	**0.016**

AUCs, area under the receiver operating characteristic curves; IR, insulin resistance; CHD, coronary heart disease; CI, Confidence intervals; CVAI, Chinese visceral adiposity index; VAI, visceral adiposity index; LAP, lipid accumulation product; TyG, triglyceride glucose; BMI, body mass index; WC, waist circumference; WHtR, waist-to-height ratio.

*P* values in bold are < 0.05.

## Discussion

The main findings of our study were as follows: Firstly, the current study demonstrated that all these seven IR surrogates (except for VAI) showed significant association with the risk of CHD incidence after fully adjustment and the subgroup analysis confirmed the associations only in participants without DM. Secondly, after taking the seven IR surrogates into account, they all showed additional predictive values in the traditional baseline risk model. Thirdly, based on the AUCs determined by ROC curves between the IR surrogates and CHD incidence, CVAI performed most favorably with the largest AUC and the differences were significant, supporting that CVAI may have better utility than other IR surrogates as a predictor to screen individuals at high risk of developing CHD. Lastly, the TyG-related indexes were better predictors for the incident CHD than the TyG index. According to our findings, CVAI and TyG-related indexes deserve attention in future research. For example, the association of CVAI and TyG-related indexes with cardiovascular risk factors or other cardiovascular diseases, such as heart failure, acute coronary syndrome should be discussed in future research. In order to broaden the application scope of CVAI and TyG-related indexes, future researches can also focus on different populations, for example, in diabetic patients or in critically ill patients.

With the development of socioeconomics and the changes of lifestyles in China, the prevalence of obesity is rising at high speed (27). According to Chinese criteria, China has the largest number of people with overweight and obesity, accounting for 34.3% and 16.4% in adults respectively (28). As a traditional independent CHD risk factors, obesity is positively related to type 2 diabetes mellitus, hypertension, and various dyslipidemias, which were generally accepted as predictors of incident CHD (29). Additionally, obesity facilitates the development of CHD partially through the positive relation of obesity to nontraditional cardiovascular risk factors, including IR, hyperinsulinemia, endothelial dysfunction, various inflammatory markers, and a variety of pro-thrombotic factors (30). Moreover, a number of meta-analyses evidenced that the TyG index, a reliable marker of IR, was closely correlated with not only traditional cardiovascular factors, such as hypertension (31) and DM (32), but also the prevalence of atherosclerotic cardiovascular diseases (33), heart failure (34, 35) and CHD (36). As a result, there were three novel TyG-related indexes directly associated with both obesity and IR indexes, that is, TyG-BMI, TyG-WC and TyG-WHtR. Although previous researches revealed that TyG-related indexes were useful IR surrogates (37) and were predictive of individuals with various cardiovascular factors (38), few studies focused on the correlation of TyG-related index with incident CHD. In addition, compared to overall obesity, fat accumulated in the abdomen was evidenced to show higher association with cardiovascular risk (39–41). Accordingly, we introduced other three IR surrogates in the current study, namely LAP, VAI and CVAI, reflecting abdominal fat deposition at the same time. Considering the vicious circle between IR and obesity, our study is the first to fill the gap and compare the seven IR surrogates mentioned above simultaneously associated with obesity in predicting the incident CHD. Moreover, we are the first to investigate whether the TyG index or the TyG-related indexes show better prediction for individuals with CHD.

CVAI was the most powerful IR surrogate in identifying participants to develop CHD among these seven indexes. Previous studies showed consistence with our findings. A cross-sectional study conducted in Shanghai including 4658 diabetic participants showed that CVAI had the highest correlation with incident CVD compared to VAI, LAP, BMI, WHR, WC, neck circumference (NC) (42). A prospective cohort study conducted in Xinjiang exhibited the same results among CVAI, VAI, LAP and WC in participants with DM (43). CVAI was also found to outperform VAI, body adiposity index (BAI), BMI, WC in predicting CHD in both non-diabetes and diabetes (44).CVAI, VAI and LAP were considered reliable marker of abdominal fat deposition (42), and increasing evidence reported that CVAI had a significant advantage in the prediction of DM (45), hypertension (46), and stroke (47). The reason contributing to this results may be that CVAI also reflects visceral adipose tissue accumulation degree and evaluates the visceral fat area for Chinese (22, 48, 49). Compared to many obesity phenotypes such as general obesity and subcutaneous fat, visceral fat showed closer correlation with cardiometabolic risk factors and is considered crucial for the prevalence of CHD (50). Our findings emphasized the value of estimating visceral fat in participants and pointed out that CVAI was a better predictor even than the widely studied IR surrogate lately, the TyG index, when metabolic measures and obesity indices were available.

BMI is generally accepted and well-established as a widely used indexes of obesity. With consideration of body fat distribution, WC and WHtR were proposed to show excellent association with abdominal adiposity (51). Previous researches supported that the three indexes of obesity may independently predict cardiovascular risk factors (40, 52). However, the question of whether the TyG-related indices were better predictors of CHD than the TyG index alone is still points of contention. Prior evidence revealed that the TyG-related index showed superiority in identifying people at high risk of cardiovascular risk factors than the TyG index, such as diabetic mellitus (38) and MetS (17). In contrast, one previous study found that the TyG index had higher predictive values for diabetic mellitus than TyG-related indices among normal-weight elderly (18). Indeed, our present study suggested that TyG-BMI, TyG-WC and TyG-WHtR had better predictive utility for CHD incidence than the TyG index. The reasons to explain may be that IR would facilitate the progress of obesity and obesity would also lead to IR due to the chronic inflammatory state. Our study made it clear that the inclusion of these obesity indices increased the ability to predict incident CHD, as opposed to the use of the TyG index alone.

In the subgroup analysis stratified by the history of DM, we conducted the multivariate Cox regression analysis to explore the associations of IR surrogates with incident CHD. We found that these positive associations with incident CHD only appeared among participants without DM. Different from our results, previous studies has evidenced the predictive value of CVAI, VAI and LAP for CVD and CHD in participants with DM (43, 44). Our explanations for the findings may be that the direct impact of DM on CHD is too great so that to conceal the influence of IR surrogates in our data. Additionally, IR showed closely associations with incident DM risk, which means diabetic patients are commonly with higher values of IR surrogates. As a result, further researches needed to be done in diabetic participants.

In conclusion, this present study included a relatively large cohort of Chinses populations. To our knowledge, this is the first study to assess CHD incidence by the seven IR surrogates simultaneously associated with obesity and compare their ability. Moreover, we firstly confirmed that TyG-related indexes surpass the TyG index in predicting CHD incidence. However, there are still several important limitations in the present study. Firstly, we did not take some cardiovascular risk factors known into account, such as family history of CHD, especially premature CHD. According to previous studies, family history of coronary heart disease (CHD) has been evidenced as an independent risk factor for CHD incidence (53). Additionally, a family history of premature coronary heart disease—before 55 years in men and 60 in women—is also a known independent risk factor for coronary artery calcification and CHD events (54). What’s worse, risk varies according to age at presentation, number of relatives affected, and degree of genetic concordance (55). Secondly, more researches are warranted to shed light on the correlations of the seven IR surrogates with CHD in populations with DM. Thirdly, as CVAI has been proposed for the Chinese population, this might be not generalizable to other populations. Finally, given the limitations of ethnic and geographic characteristics in our populations, further studies conducted in other areas are needed to test the generalization of our findings.

## Conclusion

In conclusion, all the seven IR surrogates are positively associated with the incident CHD in Chinses populations. And the combination of the TyG index and obesity measures outperform the TyG index in identifying individuals at high risk of developing CHD. Moreover, among the seven IR surrogates, the CVAI may be the best marker for developing CHD in Chinses populations.

## Data availability statement

The raw data supporting the conclusions of this article will be made available by the authors, without undue reservation.

## Ethics statement

The studies involving humans were approved by the Ethics Review Committee of the Shandong Academy of Medical Sciences. The studies were conducted in accordance with the local legislation and institutional requirements. The participants provided their written informed consent to participate in this study.

## Author contributions

LL: Conceptualization, Formal analysis, Methodology, Software, Visualization, Writing – original draft, Writing – review & editing. JP: Conceptualization, Formal analysis, Methodology, Software, Visualization, Writing – original draft, Writing – review & editing. NW: Validation, Writing – review & editing. ZW: Project administration, Resources, Writing – original draft. YZ: Writing – original draft, Project administration, Resources. HC: Formal analysis, Writing – original draft. DZ: Writing – original draft, Formal analysis. FL: Data curation, Writing – original draft, Investigation, Resources. XM: Validation, Writing – review & editing. JY: Conceptualization, Data curation, Formal analysis, Funding acquisition, Writing – original draft, Writing – review & editing.
